# Global smoking trends in inflammatory bowel disease: A systematic review of inception cohorts

**DOI:** 10.1371/journal.pone.0221961

**Published:** 2019-09-23

**Authors:** Tom Thomas, Joht Singh Chandan, Venice Sze Wai Li, Cheuk Yin Lai, Whitney Tang, Neeraj Bhala, Gilaad G. Kaplan, Siew C. Ng, Subrata Ghosh

**Affiliations:** 1 Translational Gastroenterology Unit, University of Oxford, Oxford, United Kingdom; 2 Institute of Applied Health Research, University of Birmingham, Birmingham, United Kingdom; 3 Department of Medicine and Therapeutics, Institute of Digestive Disease, State Key Laboratory of Digestive Diseases, LKS Institute of Health Science, Chinese University of Hong Kong, Hong Kong, China; 4 University Hospitals Birmingham NHS Foundation Trust, Birmingham, United Kingdom; 5 Departments of Medicine and Community Health Sciences, University of Calgary, Calgary, Alberta, Canada; 6 NIHR Biomedical Research Centre Birmingham, Institute of Translational Medicine, University of Birmingham, Birmingham, United Kingdom; Humanitas University, ITALY

## Abstract

**Background and aims:**

The effect of smoking on the risk of developing inflammatory bowel diseases (IBD) may be heterogeneous across ethnicity and geography. Although trends in smoking for the general population are well described, it is unknown whether these can be extrapolated to the IBD cohort. Smoking prevalence trends specific to the global IBD cohort over time have not been previously reported. This is a systematic review of smoking prevalence specific to the IBD cohort across geography.

**Methods:**

A systematic literature search was conducted on Medline and Embase from January 1^st^ 1946 to April 5^th^ 2018 to identify population-based studies assessing the prevalence of smoking at diagnosis in inception cohorts of Crohn’s disease(CD) or ulcerative colitis(UC). Studies that did not report smoking data from time of diagnosis or the year of IBD diagnosis were excluded. Prevalence of smoking in IBD was stratified by geography and across time.

**Results:**

We identified 56 studies that were eligible for inclusion. Smoking prevalence data at diagnosis of CD and UC was collected from twenty and twenty-five countries respectively. Never-smokers in the newly diagnosed CD population in the West has increased over the last two decades, especially in the United Kingdom and Sweden; +26.6% and +11.2% respectively. Never-smokers at CD diagnosis in newly industrialised nations have decreased over the 1990s and 2000s; China (-19.36%). Never-smokers at UC diagnosis also decreased in China; -15.4%. The former-smoker population at UC diagnosis in China is expanding; 11%(1990–2006) to 34%(2011–2013).

**Conclusion:**

There has been a reduction in the prevalence of smoking in the IBD cohort in the West. This is not consistent globally. Although, smoking prevalence has decreased in the general population of newly industrialised nations, this remains an important risk factor with longer term outcomes awaiting translation in both UC and CD.

## Introduction

Our group has extensively reported that inflammatory bowel diseases (IBD) have become a global challenge in the 21^st^ century.[1–5] The rapidly accelerating incidence of both Crohn’s disease (CD) and ulcerative colitis (UC) in the newly industrialized countries in the East mirrors epidemiological patterns of IBD in the West more than 75 years ago.[2] The evolving epidemiology of IBD is thought to be associated with the industrialisation of society. The rise of IBD incidence in newly industrialised nations combined with reports of comparable rates of IBD in migrant and native populations in the West[6] support the theory that environmental triggers and Western lifestyle have an integral role in the pathogenesis of IBD. [3,4]

The dichotomous relationship between smoking and the development of IBD has been the subject of intense scrutiny and is a complex interplay of genetics, immunology and environment. In the West, smoking has been consistently reported as a risk factor for developing CD and adversely affects disease course[7–9], whereas former smokers and non-smokers are at increased risk of developing UC in comparison to current smokers.[10,11] In contrast, studies in non-Western populations have been unable to replicate this association between CD and smoking.[12] The interaction between smoking and the NOD-2 gene and their effect on the risk of CD has been postulated to be specific to the 1007 fs mutation and a negative association between NOD-2 mutation and smoking could be explained by their inverse relationship.[13]

An understanding of global smoking prevalence trends specific to the IBD cohort is required as the foundation for further investigation of the heterogeneous influence of this risk factor in IBD pathogenesis and disease course across different regions. In addition, this is increasingly important in light of the identification of smoking as a key risk factor for non-response to anti-TNF agents in patients with CD.[14] However, the global prevalence of smoking associated with IBD have not been collated and reported. We conducted a systematic review to assess the prevalence of smoking in all population based IBD inception cohort studies. We examined smoking prevalence specific to individual IBD cohorts across time and geography.

## Materials and methods

### Search strategy and selection criteria

This systematic review was conducted according to the Meta-analysis of Observational Studies in Epidemiology (MOOSE) guidelines.[15] A systematic literature search (S1 Table) was conducted on Medline (01 January 1946 to April 5th 2018) and Embase (01 January 1947 to April 5th 2018) for studies assessing the prevalence of smoking at diagnosis in inception IBD cohorts. All studies from our previous systematic review on IBD epidemiology[1],[5] as well as the reference lists of all relevant articles were included. We also obtained data outside of the search strategy using expert knowledge of active studies as with the Asia-Pacific Crohn’s and Colitis Epidemiologic Study Group [ACCESS]).

**Fig 1:**
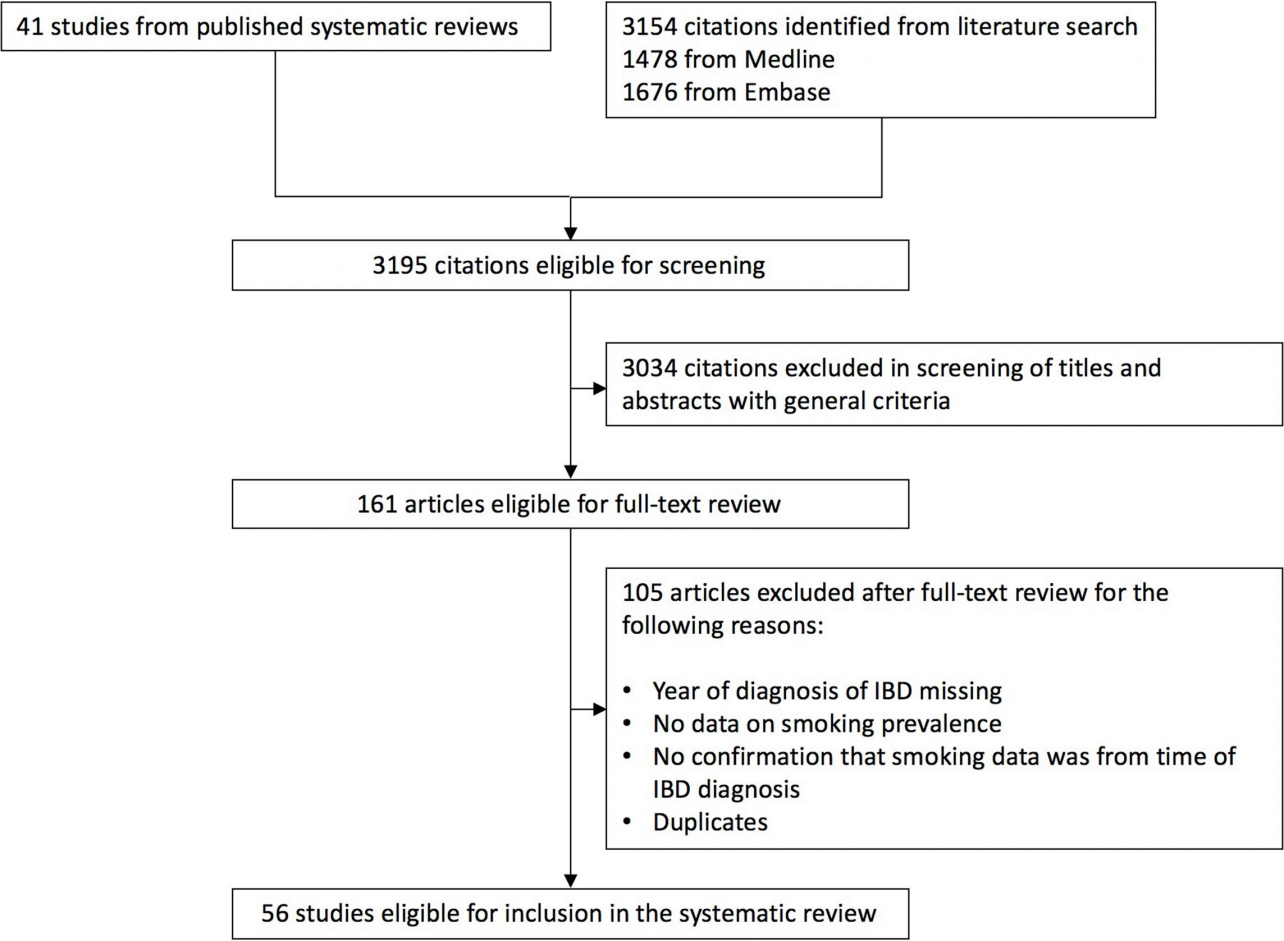
Study selection.

All stages of the systematic review were independently conducted by two teams; the first from the United Kingdom (TT and JSC) and the second from Hong Kong (SCN, VSWL and CYL). The first stage consisted of an initial screening of abstracts and titles of search results. Studies were excluded if they were not observational in design and did not report original data (i.e. review articles). Studies were considered for final inclusion in the review if their study participants consisted of a population-based inception cohort of CD and/or UC with raw numbers reported to enable the calculation of ever and/or never smoking proportion at time of IBD diagnosis. Studies could also be included if they expressed the frequency of smokers or non-smokers. A study was considered to be population-based if the sample was representative of geographical region. Smoking data had to be reported separately in CD and UC cohorts for inclusion. Studies that did not report smoking data from time of diagnosis or did not have the year of IBD diagnosis were excluded. Discrepancies between the reviewers were resolved in conjunction with GGK, SG and SCN. The flow chart for the above process is presented in Fig 1.

### Data analysis

The data extracted included: author, geographical location, study period, size of CD or UC cohort, frequency of current, former and never-smokers including unknown smoking status. Study quality was ascertained using a modified version of the Newcastle-Ottawa Scale (S2 Table). The modified scale addressed aspects of quality relevant to population-based inception cohorts as well as ascertainment of smoking exposure.

We classified geographic regions according to proximity and economic similarity based upon the United Nations classification of economic region as in our previous work.[1],[5] The regions included are: North America, South America, Eastern Europe, Northern Europe, Southern Europe, Western Europe, Asia and Oceania.

**Fig 2:**
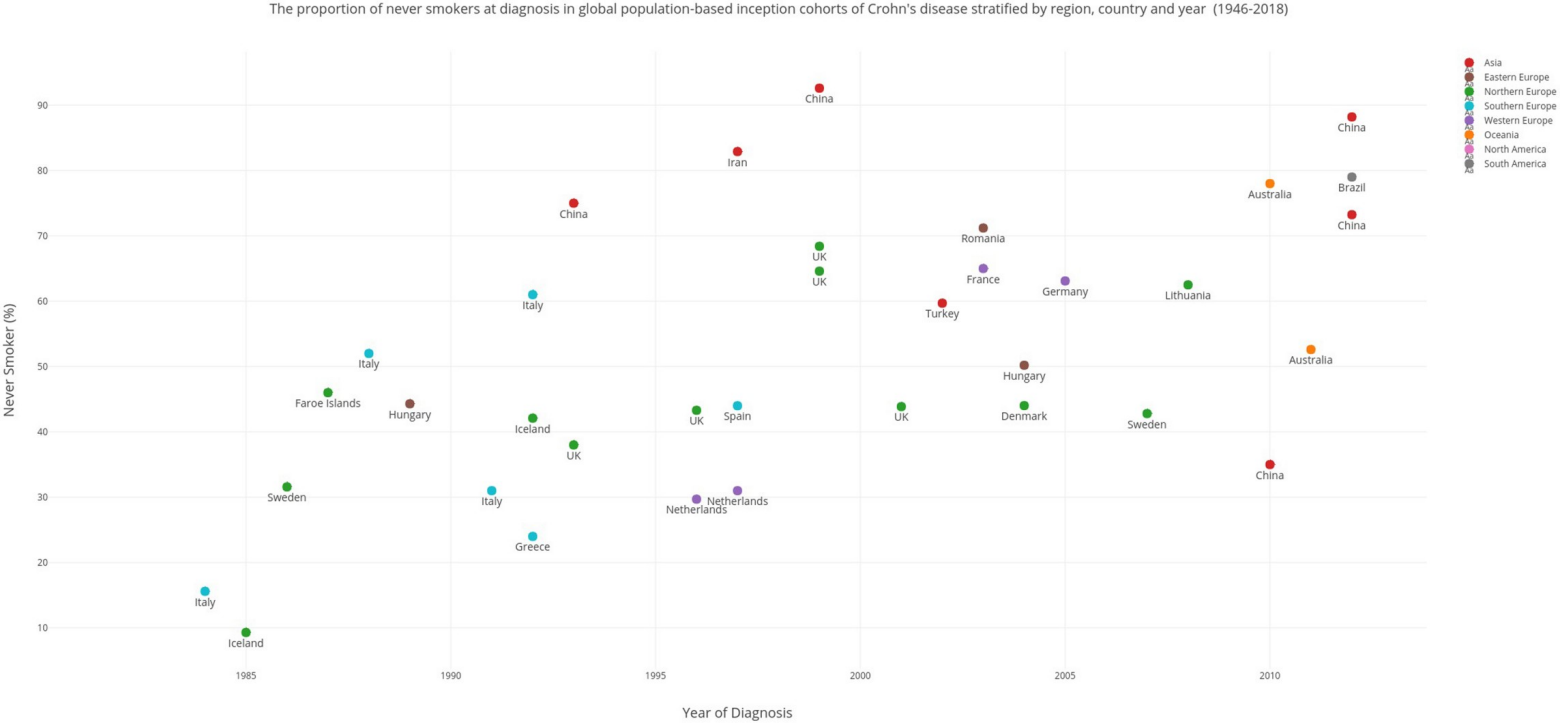
The proportion of never smokers at diagnosis in global population based inception cohorts of Crohn’s disease stratified by region, country and year (1946–2018).

**Fig 3:**
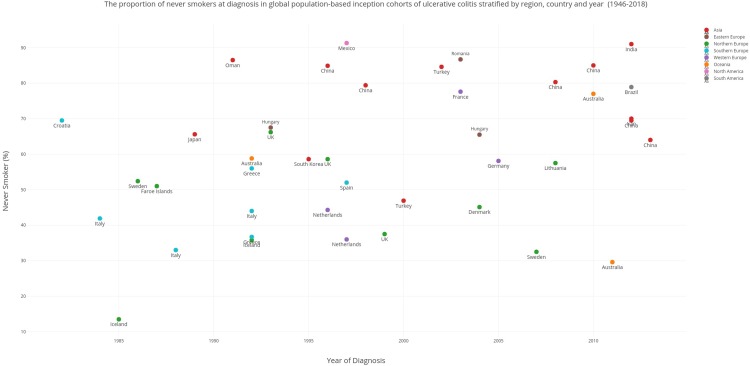
The proportion of never smokers at diagnosis in global population based inception cohorts of ulcerative colitis stratified by region, country and year (1946–2018).

Scatter plots (created using Plotly (Montreal, Canada) were used to display time trends across geography in the proportion of never and ever smokers in inception cohorts of CD (Fig 2) and UC (Fig 3) between 1980 to 2013. The earliest and latest years for which smoking data was available was 1980 and 2013 respectively. Smoking prevalence in local jurisdictions/regions were extrapolated to the entire country. In studies that reported smoking prevalence across a range of years, the median year was selected. Where studies reported former smokers, these patients were pooled with current smokers to formulate an ever-smoker category. In studies that reported only current and former category or an ever smoker category, the remainder of the population were designated as never-smokers. In UC, we sought to assess the former smoker population as this is considered the at-risk population. However, the former smoker population was also incorporated into the ever smoker population and reported for consistency. Studies with a total sample size of less than 10 subjects were excluded from these graphs. Further analysis in the form of meta-analysis or time trend analyses were not deemed appropriate due to paucity of data and heterogeneity in study design. Apart from ever smoking and never smoking data, quantitation of smoking in terms of average number of cigarettes smoked or duration of smoking were not available from the population based epidemiological data.

## Results

We identified 41 records from our previous research on IBD inception cohorts^1^. Our search strategy identified 3152 additional records from MEDLINE and Embase from January 1948 to April 31^st^ 2018. Fig 1 demonstrates the number of records eligible for and removed prior to full text review. 56 studies were eligible for final inclusion in the systematic review. These included 44 studies in CD and 46 studies in UC (Fig 1). Characteristics of all included studies are presented in Tables 1 and 2.

**Table 1:** Smoking prevalence in global population-based inception cohorts of Crohn’s disease stratified by region, country and year (1946–2018).

Author	Country	Area	Year	Total CD (n)	Age Category	Age; Mean* (SD), Median^#^ (Range)	Ever Smoker (n)	Ever Smoker (%)	Never Smoker (n)	Never Smoker (%)	Missing Data	Defined smoking groups^a^
**Asia (n = 8)**												
**Leong 2004**[56]	China	Hong Kong	1985–2001	80	all ages	33.1 (14)*	20	25	60	75	0	Yes
**Lok 2007**[30]	China	Hong Kong	1991–2006	27	all ages	26 (11–56)*	2	7.4	25	92.6	0	Yes
**Zhao 2013**[57]	China	Wuhan	2010	34	all ages	36^#^	22	65	12	35	0	Yes
**Zeng 2013**[58]	China	Guangdong	2011–2012	17	all ages	25*	2	11.8	15	88.2	0	Yes
**ACCESS Study**	China	Nationwide	2011–2013	142	-	-	38	26.8	104	73.2	3.4	Yes
**Yang 2014**[59]	China	Daqing	2012–2013	2	all ages	39.5*	2	100	0	0	0	Yes
**Zahedi 2014**[60]	Iran	Kerman	2011–2012	6	all ages	33.3*	2	33.3	4	66.6	0	No
**Tozun 2009**[61]	Turkey	Nationwide	2001–2003	216	all ages	37.4 (12.8)*	87	40.3	129	59.7	0	Yes
**Eastern Europe (n = 3)**												
**Lakatos 2013**[8]	Hungary	Veszprem	1977–2001	506	all ages	31.5 (13.8)*	239	55.7	224	44.3	0	No
**Lakatos 2011**[28]	Hungary	Veszprem	2002–2006	163	all ages	32.5 (15.1)*	81	49.8	82	50.2	0	Yes
**Gheorghe 2004**[62]	Romania	Nationwide	2002–2003	85	all ages	43.9 (15.6)*	25	29.8	60	71.2	0	Yes
**Northern Europe (n = 16)**												
**Ramadas 2010**[63]	UK	Cardiff	1986–1991	105	all ages	30 (4–78)^#^	52	49	n/a	n/a	0	No
**Yapp 2000**[18]	UK	Cardiff	1991–1995	84	all ages	-	36	43	32	38	19	Yes
**Ramadas 2010**[63]	UK	Cardiff	1992–1997	99	all ages	29 (12–73)^#^	39	39	n/a	n/a	0	No
**Garcia 2005**[64]	UK	Nationwide	1995–1997	171	20–84	-	79	46.2	74	43.3	10.5	Yes
**Tsironi 2004**[33]	UK	Tower Hamlets	1997–2001	19	all ages	19 (10–75)^#^	6	31.6	13	68.4	0	No
**Ramadas 2010**[63]	UK	Cardiff	1998–2003	137	all ages	31 (7–84)^#^	57	41	n/a	n/a	0	No
**Gunesh 2008**[65]	UK	Cardiff	1996–2005	212	all ages	31 (8–87)^#^	109	51.4	93	43.9	4.7	Yes
**Chhaya 2016**[19]	UK	Nationwide	1989–2009	9391	all ages	-	2787	29.7	6070	64.6	5.7	Yes
**Persson 1990**[16]	Sweden	Stockholm County	1984–1987	152	>15 years	-	101	66.5	48	31.6	1.97	Yes
**Sjoberg 2014**[17]	Sweden	Uppsala	2005–2009	264	all ages	34.8 (19.4)*	81	30.7	113	42.8	26.5	Yes
**Kiudelis 2012**[66]	Lithuania	Kaunas	2007–2009	16	all ages	34.94 (10.4)*	6	37.5	10	62.5	0	Yes
**Bjornsson 1998**[36]	Iceland	Nationwide	1980–1989	75	all ages	34.4 (4–79)^#^	18	24	7	9.3	66.7	Yes
**Bjornsson 2000**[37]	Iceland	Nationwide	1990–1994	64	all ages	29.7 (9–76)^#^	20	31.3	27	42.1	26.6	Yes
**Bjornsson 2015**[67]	Iceland	Nationwide	1995–2009	279	all ages	38 (3–86)^#^	76	27.2	n/a	n/a	35	No
**Hammer 2016**[68]	Faroe Islands	Faroe Islands	1960–2014	113	all ages	41*	52	54	43	46	0	Yes
**Vind 2006**[35]	Denmark	Copenhagen	2003–2005	209	all ages	31 (10–85)^#^	108	51.7	92	44	4.3	Yes
**Southern Europe (n = 9)**												
**Vucelic 1991**[69]	Croatia	Zagreb	1975–1989	106	all ages	-	52	49	n/a	n/a	n/a	No
**Manousos 1996b**[70]	Greece	Heraklion	1990–1994	37	all ages	-	28	76	9	24	0	Yes
**Franceschi 1987**[24]	Italy	Milan	1983–1984	109	all ages	-	82	75.23	17	15.6	0	Yes
**Tragnone 1993**[40]	Italy	Bologna	1986–1989	38	>10 years	36.6 (10–80)*	18	48	20	52	0	Yes
**Ranzi 1996**[25]	Italy	Crema and Cremona	1990–1994	40	all ages	-	15	39	23	61	5	Yes
**Cottone 2006**[71]	Italy	Casteltermini	1979–2002	29	all ages	29 (17–62)^#^	20	68.9	9	31	0	Yes
**Fraga 1997**[39]	Spain	Barcelona	1997	54	-	37 (14)*	30	55.5	24	44	0	Yes
**Rodrigo 2004**[27]	Spain	Oviedo	2000–2002	37	all ages	33 (15)*	17	46	n/a	n/a	n/a	No
**Garrido 2004**[26]	Spain	Huelva	1980–2003	30	all ages	32.3 (13–42)*	10	66.7	n/a	n/a	n/a	No
**Western Europe (n = 4)**												
**Abakar-Mahamat 2007**[22]	France	Corsica	2002–2003	20	all ages	29 (11–58)^#^	7	35	13	65	0	No
**Ott 2008**[23]	Germany	Oberpfalz	2004–2006	168	all ages	28.9 (1–75)^#^	62	36.9	106	63.1	0	Yes
**Van der Heide 2011**[20]	Netherlands	Leeuwarden	1996	128	≥18 years	30 (23–42)^#^	90	70.3	38	29.7	0	Yes
**Romberg-Camps 2008**[21]	Netherlands	South Limburg	1991–2002	476	all ages	34 (5–79)*	328	69	148	31	0	Yes
**Oceania (n = 2)**												
**Vegh 2014**[44]	Australia	Melbourne	2011	38	≥15 years	37 (17–77)^#^	13	34.2	20	52.6	13.2	Yes
**Niewiadomski 2015**[72]	Australia	Victoria	2007–2013	146	all ages	36 (11–82)^#^	32	22	114	78	0	No
**North America (n = 1)**												
**Edwards 2008**[73]	Barbados	Nationwide	1980–2004	47	all ages	-	2	4	n/a	n/a	n/a	No
**South America (n = 1)**												
**Parente 2015**[74]	Brazil	Piaui	2011–2012	100	≥18 years	32.9 (13.6)*	21	21	79	79	0	No

^a^The study defined the current smoker and former smoker or never smoker groups. Alternatively, the authors quantified missing data.

**Table 2:** Smoking prevalence in global population-based inception cohorts of ulcerative colitis stratified by region, country and year (1946–2018).

**Author**	**Country**	**Area**	**Year**	**Total UC (n)**	**Age Range**	Age; Mean* (SD), Median^#^ (Range)	**Ever Smoker (n)**	**Ever Smoker (%)**	**Former -Smoker (n)**	**Former -Smoker (%)**	**Never Smoker (n)**	**Never Smoker (%)**	**Missing Data (%)**	**Defined** **smoking groups^a^**
**Asia (n = 13)**														
**Chow 2009**[41]	China	Hong Kong	1985–2006	172	≥15 years	37 (12–85)^#^	26	15.1	n/a	n/a	146	84.9	0	Yes
**Lok 2008**[42]	China	Hong Kong	1990–2006	73	all ages	40.6*	15	20.6	8	11	58	79.4	0	No
**Zhai 2017**[75]	China	Yinchuan	2003–2012	421		42.7^#^	83	19.7	3	0.7	338	80.3	0	Yes
**Zhao 2013**[57]	China	Wuhan	2010	97	all ages	41 ^#^	15	16	10	10	82	85	0	Yes
**ACCESS Study**	China	Nationwide	2011–2013	334		-	96	28.7	63	18.9	232	69.5	1.8	Yes
**Yang 2014**[59]	China	Daqing	2012–2013	25	all ages	48.9 (12.5)*	9	36	5	20	16	64	0	Yes
**ACCESS Study**	India	Hyderabad	2011–2013	23		-	2	8.70	1	4.35	21	91	0	Yes
**Zahedi 2014**[60]	Iran	Kerman	2011–2012	36	all ages	39.4*	11	30	n/a	n/a	25	70	0	No
**Nakamura 1994**[76]	Japan	Nationwide	1988–1990	384	all ages	-	132	34.4	84	21.9	252	65.6	0	Yes
**Radhakrishnan 1997**[77]	Oman	Nationwide	1987–1994	108	all ages	36 (13–70)*	15	13.5	10	9.3	93	86.5	0	Yes
**Song 2018**[78]	South Korea	Seoul	1977–2014	3060	all ages	36.4 (26–48)^#^	1181	38.6	665	21.7	1794	58.6	2.8	Yes
**Tezel 2003**[79]	Turkey	Trakya	1998–2002	49	≥15 years	41 (12)*	26	53	20	40.8	23	46.9	0	Yes
**Tozun 2009**[61]	Turkey	Nationwide	2001–2003	661	all ages	42.6 (14.6)*	102	15.4	n/a	n/a	559	84.6	0	Yes
**Eastern Europe (n = 3)**														
**Lakatos 2013**[8]	Hungary	Veszprem	1977–2008	914	all ages	38.9 (15.9)*	297	32.5	161	17.6	617	67.5	0	Yes
**Lakatos 2011**[28]	Hungary	Veszprem	2002–2006	220	all ages	40.5 (17.5)*	76	34.5	46	20.9	144	65.5	0	Yes
**Gheorghe 2004**[62]	Romania	Nationwide	2002–2003	163	all ages	44.2 (14.6)*	21	13.3	n/a	n/a	142	86.7	0	Yes
**Northern Europe (n = 11)**														
**Vind 2006**[35]	Denmark	Copenhagen	2003–2005	326	all ages	38 (2–95)^#^	128	39.3	72	22.1	147	45.1	15.6	Yes
**Hammer 2016**[68]	Faroe Islands	Nationwide	1960–2014	417	all ages	41*	204	49	121	29	213	51	0	Yes
**Bjornsson 1998**[36]	Iceland	Nationwide	1980–1989	282	all ages	33.9 (11–89)^#^	53	18.8	33	11.7	38	13.5	67.7	Yes
**Bjornsson 2000**[37]	Iceland	Nationwide	1990–1994	204	all ages	34.5 (9–84)^#^	54	26.5	41	20	73	35.8	37.8	Yes
**Bjornsson 2015**[67]	Iceland	Nationwide	1995–2009	884	all ages	37 (3–91)^#^	48	5.4	n/a	n/a	n/a	n/a	51	No
**Kiudelis 2012**[66]	Lithuania	Kaunas	2007–2009	87	>15 years	50 (17)*	37	42.5	25	28.7	50	57.5	0	Yes
**Persson 1990**[16]	Sweden	Stockholm County	1984–1987	145	15–79	-	69	47.6	26	17.9	76	52.4	0	Yes
**Sjoberg 2013**[34]	Sweden	Uppsala	2005–2009	526	all ages	39.2 (19.3)*	171	32.5	130	24.7	171	32.5	35	Yes
**Carr 1999**[31]	UK	Leicester	1991–1994	74	≥16 years	-	25	33.8	16	21.6	49	66.2	0	Yes
**Garcia 2005**[32]	UK	Nationwide	1995–1997	222	20–84	-	64	28.9	27	12.2	130	58.6	12.6	Yes
**Tsironi 2004**[33]	UK	Tower Hamlets	1997–2001	16	all ages	28 (11–73)^#^	10	62.5	3	18.8	6	37.5	0	No
**Author**	**Country**	**Area**	**Year**	**Total UC (n)**	**Age Range**	Age; Mean* (SD), Median^**#**^ **(Range)**	**Ever Smoker (n)**	**Ever Smoker (%)**	**Former -Smoker (n)**	**Former -Smoker (%)**	**Never Smoker (n)**	**Never Smoker (%)**	**Missing Data (%)**	**Defined** **smoking groups**^**a**^
**Southern Europe (n = 9)**														
**Vucelic 1991**[80]	Croatia	Zagreb	1975–1989	265	all ages	-	81	30.5	51	19.2	184	69.5	0	No
**Ladas 2005**[81]	Greece	Trikala	1990–1994	66	≥10 years	-	29	44	16	24.3	37	56	0	Yes
**Manousos 1996a**[82]	Greece	Heraklion	1990–1994	117	all ages	-	74	63.3	60	51.3	43	36.7	0	No
**Franceschi 1987**[24]	Italy	Milan	1983–1984	124	all ages	-	72	58.1	46	37.1	52	41.9	0	Yes
**Tragnone 1993**[40]	Italy	Bologna	1986–1989	73	>10 years	44.2 (16–74)^#^	49	68	31	43	24	33	0	Yes
**Ranzi 1996**[25]	Italy	Crema and Cremona	1990–1994	82	all ages	-	45	56	32	39	35	44	2.4	Yes
**Garrido 2004**[26]	Spain	Huelva	1980–2003	40	all ages	44.7 (39–51)*	5	12.5	n/a	n/a	n/a	n/a	n/a	No
**Fraga 1997**[39]	Spain	Barcelona	1997	86	-	40 (15)*	41	48	18	21	45	52	0	Yes
**Rodrigo 2004**[27]	Spain	Oviedo	2000–2002	47	all ages	45 (20)*	19	40	n/a	n/a	n/a	n/a	n/a	No
**Western Europe (n = 4)**														
**Abakar-Mahamat 2007**[22]	France	Corsica	2002–2003	49	all ages	44 (18–80)^#^	11	22.5	8	16.33	38	77.6	0	No
**Ott 2008**[23]	Germany	Oberpfalz	2004–2006	105	all ages	39.5 (7–81)^#^	44	41.9	32	30.5	61	58.1	0	Yes
**Van der Heide 2011**[20]	Netherlands	Leeuwarden	1996	192	≥18 years	35 (27–50)^#^	107	55.8	60	31.3	85	44.3	0	Yes
**Romberg-Camps 2008**[21]	Netherlands	South Limburg	1991–2002	630	all ages	42 (8–84)*	403	64	277	44	227	36	0	Yes
**Oceania (n = 3)**														
**Abraham 2003**[43]	Australia	Sydney	1990–1993	102	all ages	-	42	41.2	30	29.4	60	58.8	0	Yes
**Vegh 2014**[44]	Australia	Melbourne	2011	27	≥15 years	40 (17–87)^#^	11	40.7	10	37	8	29.6	n/a	Yes
**Niewiadomski 2015**[72]	Australia	Victoria	2007–2013	96	all ages	40 (11–87)^#^	22	23	17	18	74	77	0	No
**North America (n = 2)**														
**Edwards 2008**[73]	Barbados	Nationwide	1980–2004	121	all ages	-	3	2	n/a	n/a	n/a	n/a	n/a	No
**Yamamoto-Furusho 2009**[83]	Mexico	Mexico City	1987–2006	848	all ages	31.3 (12.3)*	73	8.6	73	8.6	775	91.3	0	Yes
**South America (n = 1)**														
**Parente 2015**[74]	Brazil	Piaui	2011–2012	152	≥18 years	36.8 (14.8)*	32	21.1	n/a	n/a	120	78.9	0	No

^a^The study defined the current smoker, former smoker or never smoker groups. Alternatively, the authors quantified missing data.

Smoking prevalence figures were reported for: North America (2 studies), South America (1 study), Eastern Europe (3 studies), Northern Europe (16 studies), Southern Europe (12 studies), Western Europe (4 studies), Asia (15 studies), and Oceania (3 studies). Scatter plots representing never-smoker prevalence in the CD and UC cohorts from 1980 to 2018 stratified by geographic region are presented in Figs 2 and 3 respectively. Smoking prevalence varied greatly according to geographic region. Fig 2 shows that an increasing number of the newly diagnosed CD population over the last two decades in the West particularly in the UK have never smoked. In contrast, a decrease in the proportion of never-smokers over the 1990s and 2000s is seen in newly industrialised nations such as China. Fig 3 is suggestive of significant heterogeneity in the trend of the never-smoker group in the newly diagnosed UC population in the West. Data from the United Kingdom and Sweden over the 1980s and 1990s suggest a decrease in this group whilst data from Iceland and Italy show an increase in the never-smoking proportion. Fig 3 shows that the proportion of people who have never smoked at UC diagnosis in newly industrialised nations particularly China has been decreasing over the last two decades. Tables 3 and 4 displays these ranges stratified according to geographic region.

**Table 3:** Prevalence of never-smokers in global population-based inception cohorts of Crohn’s disease and ulcerative colitis stratified by range and region (1946–2018).

	Crohn’s disease		Ulcerative colitis	
Region	Lowest estimate	Highest estimate	Lowest estimate	Highest estimate
**North America**	n/a	n/a	n/a	91.3% (n = 848) 1987–2006; Mexico City, Mexico
**South America**	n/a	79% (n = 100) 2011–2012; Piaui, Brazil	n/a	78.9% (n = 152) 2011–2012; Piaui, Brazil
**Eastern Europe**	44.3% (n = 506) 1977–2001; Veszprem, Hungary	71.20% (n = 85) 2002–2003; Nationwide, Romania	65.5% (n = 220) 2002–2006; Veszprem, Hungary	86.7% (n = 163) 2002–2003; Nationwide, Romania
**Northern Europe**	9.3% (n = 75) 1980–1989; Nationwide, Iceland	68.4% (n = 19) 1997–2001; Tower Hamlets London, United Kingdom	13.5% (n = 282) 1980–1989; Nationwide, Iceland	66.2% (n = 74) 1991–1994; Leicester, United Kingdom
**Southern Europe**	15.6% (n = 109) 1983–1984; Milan, Italy	61% (n = 40) 1990–1994; Crema and Cremona, Italy	33% (n = 73) 1986–1989; Bologna, Italy	69.5% (n = 265) 1975–1989; Zagreb, Croatia
**Western Europe**	29.7% (n = 128) 1996; Leeuwarden, Netherlands	65% (n = 20) 2002–2003; Corsica, France	36% (n = 630) 1991–2002; South Limburg, Netherlands	77.6% (n = 49) 2002–2003; Corsica, France
**Asia**	35% (n = 34) 2010; Wuhan, China	92.6% (n = 27) 1991–2006; Hong Kong, China	46.9% (n = 49) 1998–2002; Trakya, Turkey	91% (n = 23) 2011–2013; Hyderabad, India, India
**Oceania**	52.6% (n = 38) 2011; Melbourne, Australia	78% (n = 32) 2007–2013; Victoria, Australia	29.6% (n = 27) 2011; Melbourne, Australia	77% (n = 96) 2007–2013; Victoria, Australia

N: total cohort size; n/a: not available; Studies with n<10 have been excluded.

**Table 4:** Prevalence of ever-smokers in global population-based inception cohorts of Crohn’s disease and ulcerative colitis stratified by range and region (1946–2018).

	Crohn’s disease		Ulcerative colitis	
Region	Lowest estimate	Highest estimate	Lowest estimate	Highest estimate
**North America**	n/a	4% (n = 47) 1980–2004; Barbados, Nationwide	n/a	8.6% (n = 848) 1987–2006; Mexico City, Mexico
**South America**	n/a	21% (n = 100) 2011–2012; Piaui, Brazil	n/a	21.1% (n = 152) 2011–2012; Piaui, Brazil
**Eastern Europe**	29.8% (n = 85) 2002–2003; Nationwide, Romania	55.7% (n = 506) 1977–2001; Veszprem, Hungary	13.3% (n = 163) 2002–2003; Nationwide, Romania	34.5% (n = 220) 2002–2006; Veszprem, Hungary
**Northern Europe**	24% (n = 75) 1980–1989; Nationwide, Iceland	66.5% (n = 152) 1984–1987; Stockholm County, Sweden	5.4% (n = 884) 1995–2009; Nationwide, Iceland	62.5% (n = 16) 1997–2001; Tower Hamlets, United Kingdom
**Southern Europe**	39% (n = 40) 1990–1994; Crema and Cremona, Italy	76% (n = 37) 1990–1994; Heraklion, Greece	12.5% (n = 40) 1980–2003; Huelva, Spain	68% (n = 73) 1986–1989; Barcelona, Spain
**Western Europe**	35% (n = 20) 2002–2003; Corsica, France	70.3% (n = 128) 1996; Leeuwarden, Netherlands	22.5% (n = 49) 2002–2003; Corsica, France	64% (n = 630) 1991–2002; South Limburg, Netherlands
**Asia**	7.4% (n = 27) 1991–2006; Hong Kong, China	65% (n = 34) 2010; Wuhan, Turkey	8.70% (n = 23) 2012; Hyderabad, India	53% (n = 49) 1998–2002; Trakya, Turkey
**Oceania**	22% (n = 146) 2007–2013; Victoria, Australia	34.2% (n = 38) 2011; Melbourne, Australia	23% (n = 96) 2007–2013; Victoria, Australia	41.2% (n = 102) 1990–1993; (Sydney, Australia)

N: total cohort size; n/a: not available; Studies with n<10 have been excluded.

### Crohn’s disease

Smoking prevalence data at diagnosis of CD was collected from twenty countries. The western world particularly Europe has demonstrated an overall increase in the prevalence of never smokers in the newly diagnosed CD cohort over the last three decades.

In Sweden (Northern Europe), the proportion of never-smokers increased from 31.6%[16] in the 1980s to 42.8% (2007)[17]. In the early 1990s, the proportion of never-smokers in the newly diagnosed CD cohort in the UK was 38%.[18] A large population-based inception cohort study (1989–2009)[19] in the UK estimated that 64.6% of newly diagnosed CD patients were never-smokers. This trend is replicated in Western Europe, Southern Europe and Eastern Europe. The proportion of never-smokers in the CD cohort in the Netherlands ranged from 29.7%[20] to 31%[21] in the 1990s however France and Germany demonstrated a never-smoker proportion of 65%[22] and 63.1%[23] in the 2000s respectively. In Italy (Southern Europe), there was a steady increase in the never-smoker population at CD diagnosis over the course of the 1980s[24] and 1990s.[25] Similarly, Spain showed consistent trends with the ever-smoker group steadily declining from 66%(1980 and 1990s)[26] to 46%(2001)[27] in the newly-diagnosed CD cohort. Similarly, in Hungary (Eastern Europe), the proportion of never smokers in the newly diagnosed CD cohort increased from 44.3%[8] to 50.2%[28] over the course of 30 years.

In contrast to Europe, smoking prevalence in inception CD cohorts in Asia appears to be increasing over time. The majority of CD subjects in Asia were never-smokers. The proportion of subjects who had never smoked range from 75%[29] (Hong Kong, China;1985–2001) to 92.6%[30] (Hong Kong, China;1991–2006). However, in a more recent inception cohort from Asia from 2011-2013(ACCESS), 73.2% of CD subjects were never smokers. Nine out of 44 studies did not report former smokers. Never-smoker populations were assumed to be the remainder of the population if ever smoker data was provided.

### Ulcerative colitis

Smoking prevalence data at diagnosis of UC was collected from twenty-five countries. Smoking trends in Europe for the newly diagnosed UC cohort showed more heterogeneity than in CD. Data from the United Kingdom appear to suggest a decrease in the never-smoker proportion in the UC cohort during the 1990s; 66.2%(1993)[31] to 58.6%(1995–1997)[32] and 37.5% (1999)[33]. The former smoker population appears to have decreased in the same decade from 21.6%[31] to 18.8%[33]. Data from Sweden demonstrate reduction in the never smoker population from 52.4%(1984–1987)[16] to 32.5%(2005–2009)[34]. An increase in the proportion of former smokers at diagnosis from 17.9%(1984–1987)[16] to 24.7%(2005–2009)[34] was noted. Other Scandinavian regions such as Denmark showed only a slightly higher proportion of never smokers in their UC cohorts; 45%(2004)[35]. In contrast to the remainder of Northern Europe in the 1980s and 1990s, Iceland demonstrated an increase in the never-smoker proportion from 13.5%[36] to 35.8%[37] across this period. The percentage of former smokers at UC diagnosis also rose from 11.7% to 20% across those two decades. These results co-relate with a decrease in the ever-smoker proportion down to 48%[38] in the 21^st^ century.

In Southern Europe, Spain demonstrated an increase in their ever-smoker proportion from 12.5%[26] in the 1980s and 1990s to 48%[39] in the 2000s. In Italy, the never-smoker proportion varied by geographic region; 42% (Milan; early 1980s)[24] to 33% (Bologna; late 1980s)[40] and 44% (Crema and Cremona; early 1990s)[25]. Former-smoker proportions were similarly varied across these regions and time periods: 37.1%, 43% and 39% respectively. In Eastern Europe, Veszprem (Hungary) demonstrated a reduction in the never-smoker population in the UC cohort; 67.5%(1977–2008)[8] to 65.5%(2002–2006)[28]. The former smoker population in the newly diagnosed UC cohort during those periods were 17.6% and 20.9% respectively. In contrast to the rest of Europe, never-smoker proportions increased over the late 1990s and early 2000s in Western Europe from 44.3%[20] (Netherlands; 1996) to 77.55% (France; 2003)[22] and 58.09%(Germany;2005)[23].

The proportion of newly diagnosed UC subjects who have never smoked has decreased in China over the last two decades. The proportion who had never smoked were 84.9%[41] in China in the late 1990s. By 2012 these figures had decreased to 69.5% (ACCESS Cohort;2011–2013). The proportion of former smoker patients in the newly diagnosed UC cohort in China appears to be increasing from 11%(1990–2006)[42] to 34% (Hong Kong ACCESS cohort).

Data from major cities in Australia suggest that the proportion of never-smokers in the newly diagnosed UC cohort has increased from 58.8%(Sydney;1992)[43] to 77%(Victoria; 2007–2013)[44]. The former smoker proportion of patients at diagnosis also decreased from 29.4% to 18% across the same regions and time periods respectively. The impact of missing smoking data regarding the participants vary due to heterogeneity in reporting. Four out of the 46 studies included for UC did not report former smokers at diagnosis. Never-smoker populations were assumed to be the remainder of the population if ever smoker data was provided.

## Discussion

We present a comprehensive review of smoking trends over time in inception IBD cohorts worldwide. In the West, the proportion of newly diagnosed CD subjects who have never smoked has increased over time. The proportion of newly diagnosed UC subjects who have never smoked has declined in the 1980s and 1990s in Europe although an increase was noted in Western Europe from the late 1990s. In contrast, the proportion of subjects who have never smoked at IBD diagnosis has decreased in Asia, particularly in China. Thus, we demonstrate that trends in smoking prevalence specific to the IBD cohort do not mirror global trends in smoking discerned from the general population.[45]

The incidence of IBD in newly industrialised countries is accelerating whilst the incidence of IBD is stabilising in the West.[1] The effect of smoking on the incidence of IBD across the globe likely varies due to heterogeneity in genetic susceptibility and the presence of other risk factors. Public health measures in the 1980s and 1990s led to a reduction of smoking prevalence in the general population in many Western countries.[46] The higher proportion of never smokers at diagnosis of CD over time may be explained by adolescents who decided not to smoke in the 1990s. This could have potentially contributed to the stabilization, and in certain regions decrease, in the incidence of adult-onset CD in some Western countries. This ecological trend could also explain the decrease in the former smoker population in the UK over the 1990s and could have contributed to the recent stabilisation of UC incidence.[1]

In contrast, we are at the infancy of the IBD ‘epidemic’ in newly industrialized countries in Asia, especially in areas of high smoking prevalence[46]; hence ever-smoker trends at IBD diagnosis are on the increase. The rapid expansion of the former smoker population at UC diagnosis in China is suggestive of a rapid expansion of the at-risk population. The Global Burden of Disease Study 2015 identified China as one of the leading countries in the world for the total number of smokers.[46] In line with the Lopez model[47], these newly industrialised nations are rapidly moving towards Stage IV where smoking prevalence in the general population will decrease as societal attitudes shift and government anti-smoking policy becomes comprehensive. This could potentially foreshadow a protracted course of high UC incidence in comparison to CD. Similar to the West, we hypothesise that a ‘lag effect’ can be expected in future epidemiological studies particularly in CD based in newly industrialised nations. However, due to the complex interplay between genetics and environment in the development of IBD, this effect may not be as pronounced as in the West[12] although in contrast to CD, there is some evidence to suggest that the role of smoking in UC is uniform across the East and West.[12,48,49]

The concurrent decrease of the never-smoker population in both CD and UC cohorts in newly industrialised nations is potentially suggestive of significant heterogeneity in the interaction between smoking and the process of IBD development across geographic regions. Even in the West, the incidence of CD had been high in relatively low smoking prevalence populations i.e. Israeli Jews[50], Canada, and Sweden. Multiple studies[12],[29],[51] in the Asia-Pacific region have demonstrated that active smoking does not confer an increased risk of CD in this population as it does in the West. The relative absence of the NOD-2 mutation in CD cases in Japan suggests that the role of smoking in IBD is subject to underlying genetic heterogeneity.[52] Environmental factors such as air pollution[53], diet and a Western lifestyle as demonstrated in migrant sub-populations[6] as well as evolving early life feeding patterns and improved hygiene as part of socioeconomic development[12] could be more potent mediators of IBD development.[54,55]

Our study has several limitations predominantly due to lack of available data. We were unable to perform a meta-analysis or ecological trend analysis due to study heterogeneity. Small sample sizes in some studies have also increased the risk of imprecise estimates for smoking prevalence. A paucity of gender-specific, age-category specific smoking prevalence data, data relating to quantification of smoking habits or breakdown of rural vs. urban data in the IBD cohort did not allow for further sub-group analysis. Although, it is possible studies that included children and adolescents would have a higher prevalence of never-smokers, summary statistics from included studies suggest this is not the case. The exposure to smoking was reported inconsistently; some studies reported current and former smokers whilst others reported ever and never smokers. Twenty three out of ninety-five included cohorts reported missing smoking data on participants (Tables 1 and 2). No data was available regarding second-hand smoking exposure. Due to differing study periods and the generalisation of regions to represent countries, smoking data was not fully homogenous. The inequalities in healthcare access across the globe can also affect data collection and reporting. In addition, we acknowledge that the attributable risk of smoking on IBD is low (i.e. most IBD patients do not have a history of smoking [current or former] prior to their diagnosis), however it remains an important risk mediator in the development of IBD.

Despite these limitations, this study provides a comprehensive overview of the prevalence of smoking in the global IBD cohort across time and geography. The proportion of never-smokers in IBD cohorts from newly industrialised countries appears to be decreasing over time in contrast to the IBD cohorts in the West. In light of our previous work and this study, it appears that IBD epidemiological patterns globally can be modelled along geographical and development lines within a context of genetic heterogeneity and environmental ecological exposures. It remains of clinical importance for medical practitioners to record information and act on smoking status for patients with IBD regardless of geography and ethnicity, especially in light of data suggesting smoking confers an adverse disease course in CD and is a risk factor in non-response to anti-TNF therapy.[14] Large-scale prospective inception cohorts assessing the associations of smoking for both UC and CD in Eastern and Western populations will add to the available data.

This is the first systematic review to assess trends in the prevalence of smoking in the IBD cohort worldwide. It provides a foundation for future work assessing the prevalence of this important risk mediator in a global setting as well as highlighting some of the challenges surrounding this data. A deeper understanding of IBD aetiology in relation to diet and other environmental factors across geographic regions and ethnicities is urgently required in order to formulate strategies to slow the global increase in the incidence of IBD.

## Supporting information

S1 Table Detailed MEDLINE and EMBASE search strategy for article selection (1 January 1947 to April 5 2018). (DOCX)

S2 Table Quality assessment of manuscripts (modified Newcastle Ottawa scale). (DOCX)

## Abbreviations

(IBD): Inflammatory Bowel Disease
(CD): Crohn’s disease
(UC): ulcerative colitis
