# Supplementary material for: Global smoking trends in inflammatory bowel disease: A systematic review of inception cohorts
Source: PLoS One. 2019 Sep 23;14(9):e0221961. doi: 10.1371/journal.pone.0221961 (PMC6756556; doi:10.1371/journal.pone.0221961)
Supplement: S1 Table — (DOCX) [file pone.0221961.s001.docx]

**S1 Table: Detailed MEDLINE and EMBASE search strategy for article selection (1 January 1947 to April 5 2018)**

| **MEDLINE** | **EMBASE** |
| --- | --- |
| 1. Inflammatory Bowel Diseases/ 2. Colitis, Ulcerative/ 3. Crohn Disease/ 4. Epidemiology/ 5. epidemiolog*.tw. 6. (crohn* or (ulcerative adj5 colitis) or inflammatory bowel*).tw. 7. smok* 8. Tobacco* 9. incidence.mp. or *Incidence/ 10. 1 or 2 or 3 or 4 or 5 or 6 or 9 11. 7 or 8 12. 10 and 11 | 1. *Crohn disease/ 2. *ulcerative colitis/ 3. Epidemiology/ 4. epidemiolog*.tw. 5. (inflammatory bowel disease* or IBD or crohn or (ulcerative adj5 colitis)).tw. 6. incidence.mp. or *Incidence/ 7. 1 or 2 or 3 or 4 or 5 or 6 8. Smok* 9. Tobacco* 10. 8 or 9 11. 7 and 10 |
