# Supplementary material for: Global smoking trends in inflammatory bowel disease: A systematic review of inception cohorts
Source: PLoS One. 2019 Sep 23;14(9):e0221961. doi: 10.1371/journal.pone.0221961 (PMC6756556; doi:10.1371/journal.pone.0221961)
Supplement: S2 Table — (DOCX) [file pone.0221961.s002.docx]

Supplementary Material

**S2 Table: Quality Assessment of manuscripts (modified Newcastle Ottawa Scale)**

| **First Author (Publication Year)** | **Cohort/CC** | **Is the patient definition adequate*** | **Representativeness of the population**** | **Ascertainment of exposure*** |
| --- | --- | --- | --- | --- |
| Abakar-Mahamat 2007^1^ | C | a | a | d |
| Abraham 2003^2^ | CC | a | a | b |
| ACCESS Study 2012 (China) | C | a | a | c |
| ACCESS Study 2012 (India) | C | a | a | c |
| Bjornsson 1998^3^ | C | a | a | a |
| Bjornsson 2000^4^ | C | a | a | d |
| Bjornsson 2015^5^ | C | a | a | d |
| Carr 1999^6^ | C | a | b | b |
| Chhaya 2016^7^ | C | b | a | a |
| Chow 2009^8^ | C | a | a | a |
| Cottone 2006^9^ | C | a | a | b |
| Edwards 2008^10^ | C | a | a | a |
| Fraga 1997^11^ | CC | a | b | a |
| Franceschi 1987^12^ | CC | b | a | a |
| Garcia 2005^13^ | C | a | b | a |
| Garrido 2004^14^ | C | a | a | d |
| Gheorghe 2004^15^ | C | a | b | d |
| Gunesh 2008^16^ | C | a | a | a |
| Hammer 2016^17^ | C | a | a | a |
| Kiudelis 2012^18^ | C | a | a | d |
| Ladas 2005^19^ | C | a | a | d |
| Lakatos 2011^20^ | C | a | a | a |
| Lakatos 2013^21^ | C | a | a | b |
| Leong 2004^22^ | C | a | a | d |
| Lok 2007^23^ | C | a | a | a |
| Lok 2008^24^ | C | a | a | a |
| Manousos 1996a^25^ | C | a | a | d |
| Manousos 1996b^26^ | C | a | a | d |
| Nakamura 1994^27^ | CC | b | b | a |
| Niewiadomski 2015^28^ | C | b | a | d |
| Ott 2008^29^ | C | a | a | d |
| Parente 2015^30^ | C | a | b | d |
| Persson 1990^31^ | CC | a | a | a |
| Radhakrishnan 1997^32^ | C | a | a | d |
| Ramadas 2010^33^ | C | a | a | a |
| Ranzi 1996^34^ | C | a | a | d |
| Rodrigo 2004^35^ | C | a | a | d |
| Romberg-Camps 2008^36^ | C | a | a | d |
| Sjoberg 2013^37^ | C | a | a | a |
| Sjoberg 2014^38^ | C | a | a | d |
| Song 2018^39^ | C | b | a | a |
| Tezel 2003^40^ | C | a | a | d |
| Tozun 2009^41^ | C | a | b | d |
| Tragnone 1993^42^ | C | b | a | b |
| Tsironi 2004^43^ | C | a | b | a |
| Van der Heide 2011^44^ | C | a | b | c |
| Vegh 2014^45^ | C | a | a | d |
| Vind 2006^46^ | C | a | a | a |
| Vucelic 1991^47^ | C | a | a | d |
| Yamamoto-Furusho 2009^48^ | C | a | a | a |
| Yang 2014^49^ | C | a | b | d |
| Yapp 2000^50^ | C | a | a | d |
| Zahedi 2014^51^ | C | a | a | b |
| Zeng 2013^52^ | C | a | a | d |
| Zhai 2017^53^ | C | b | b | a |
| Zhao 2013^54^ | C | a | a | d |

**References:**

1 Abakar-Mahamat A, Filippi J, Pradier C, Dozol A, Hébuterne X. Incidence of inflammatory bowel disease in Corsica from 2002 to 2003. *Gastroenterol Clin Biol* 2007; **31**: 1098–1103.

2 Abraham N, Selby W, Lazarus R, Solomon M. Is smoking an indirect risk factor for the development of ulcerative colitis? An age- and sex-matched case-control study. *J Gastroenterol Hepatol* 2003; **18**: 139–146.

3 S. BJÖRNSSON, J. H. JOHANNSSON, E. JHJEO. Inflammatory Bowel Disease in Iceland, 1980-89: A Retrospective Nationwide Epidemiologic Study. *Scand J Gastroenterol* 1998; **33**: 71–77.

4 JH BS and J. Inflammatory bowel disease in Iceland, 1990-1994: a prospective, nationwide, epidemiological study.pdf. *Eur J Gastroenterol Hepatol* 2000; **12**: 31–38.

5 Björnsson S, Tryggvason FP, Jónasson JG, Cariglia N, Örvar K, Kristjánsdóttir S *et al.* Incidence of inflammatory bowel disease in Iceland 1995 - 2009. A nationwide population-based study. *Scand J Gastroenterol* 2015; **50**: 1368–1375.

6 Carr I, Mayberry JF. The effects of migration on ulcerative colitis: A three-year prospective study among Europeans and first- and second-generation South Asians in Leicester (1991-1994). *Am J Gastroenterol* 1999; **94**: 2918–2922.

7 Chhaya V, Saxena S, Cecil E, Subramanian V, Curcin V, Majeed A *et al.* Emerging trends and risk factors for perianal surgery in Crohn’s disease. *Eur J Gastroenterol Hepatol* 2016; **28**: 890–895.

8 Chow DKL, Leong RWL, Tsoi KKF, Ng SSM, Leung W, Wu JCY *et al.* Long-term Follow-up of Ulcerative Colitis in the Chinese Population. *Am J Gastroenterol* 2009; **104**: 647–654.

9 Cottone M, Renda MC, Mattaliano A, Oliva L, Fries W, Criscuoli V *et al.* Incidence of Crohn’s disease and CARD15 mutation in a small township in Sicily. *Eur J Epidemiol* 2006; **21**: 887–892.

10 Edwards CN, Griffith SG, Hennis AJ, Hambleton IR. Inflammatory bowel disease: Incidence, prevalence, and disease characteristics in Barbados, West Indies. *Inflamm Bowel Dis* 2008; **14**: 1419–1424.

11 Fraga XF, Vergara M, Medina C, Casellas F, Bermejo B MJ. Effects of smoking on the presentation and clinical course of inflammatory bowel disease.pdf. *Eur J Gastroenterol Hepatol* 1997; **9**: 683–7.

12 Franceschi S, Panza E, Vecchia C La, Parazzini F, Decarli A, Porro GB. Nonspecific inflammatory bowel disease and smoking. *Am J Epidemiol* 1987; **125**: 445–452.

13 GARCIA RODRIGUEZ LA, GONZALEZ-PEREZ A, JOHANSSON S, WALLANDER M-A. Risk factors for inflammatory bowel disease in the general population. *Aliment Pharmacol Ther* 2005; **22**: 309–315.

14 Garrido a, Martínez MJ, Ortega J a, Lobato a, Rodríguez MJ, Guerrero FJ. Epidemiology of chronic inflammatory bowel disease in the Northern area of Huelva. *Rev Esp Enferm Dig* 2004; **96**: 687–91; 691–4.

15 Gheorghe C, Pascu O, Gheorghe L, Iacob R, Dumitru E, Tantau M *et al.* Epidemiology of inflammatory bowel disease in adults who refer to gastroenterology care in Romania: A multicentre study. *Eur J Gastroenterol Hepatol* 2004; **16**: 1153–1159.

16 Gunesh S, Thomas GAO, Williams GT, Roberts A, Hawthorne AB. The incidence of Crohn’s disease in Cardiff over the last 75 years: An update for 1996-2005. *Aliment Pharmacol Ther* 2008; **27**: 211–219.

17 Hammer T, Nielsen KR, Munkholm P, Burisch J, Lynge E. The Faroese IBD study: Incidence of inflammatory bowel diseases across 54 years of population-based data. *J Crohn’s Colitis* 2016; **10**: 934–942.

18 Kiudelis G, Jonaitis L, Adamonis K, Žvirbliene A, Tamelis A, Kregždyte R *et al.* Incidence of inflammatory bowel disease in kaunas region, lithuania. *Med* 2012; **48**: 431–435.

19 Ladas SD, Mallas E, Giorgiotis K, Karamanolis G, Trigonis D, Markadas A *et al.* Incidence of ulcerative colitis in Central Greece: A prospective study. *World J Gastroenterol* 2005; **11**: 1785–1787.

20 Lakatos L, Kiss LS, David G, Pandur T, Erdelyi Z, Mester G *et al.* Incidence, disease phenotype at diagnosis, and early disease course in inflammatory bowel diseases in Western Hungary, 2002-2006. *Inflamm Bowel Dis* 2011; **17**: 2558–2565.

21 Lakatos PL, Vegh Z, Lovasz BD, David G, Pandur T, Erdelyi Z *et al.* Is Current smoking still an important environmental factor in inflammatory bowel diseases? Results from a population-based incident cohort. *Inflamm Bowel Dis* 2013; **19**: 1010–1017.

22 R.W.L. L, J.Y. L, Leong RWL, Lau JY, Sung JJY. The epidemiology and phenotype of Crohn’s disease in the Chinese population. *Inflamm Bowel Dis* 2004; **10**: 646–651.

23 Lok KH, Hung HG, Ng CH, Li KK, Li KF, Szeto ML. The epidemiology and clinical characteristics of Crohn’s disease in the Hong Kong Chinese population: experiences from a regional hospital. *Hong Kong Med J* 2007; **13**: 436–41.

24 Lok K-H, Hung H-G, Ng C-H, Kwong KC, Yip W-M, Lau S-F *et al.* Epidemiology and clinical characteristics of ulcerative colitis in Chinese population: Experience from a single center in Hong Kong. *J Gastroenterol Hepatol* 2008; **23**: 406–410.

25 Manousos ON, Koutroubakis I, Potamianos S, Roussomoustakaki M, Gourtsoyiannis N, Vlachonikolis IG. A prospective epidemiologic study of Crohn’s disease in Heraklion, Crete: Incidence over a 5-year period. *Scand J Gastroenterol* 1996; **31**: 599–603.

26 Manousos ON, Giannadaki E, Mouzas IA, Tzardi M, Koutroubakis I, Skordilis P *et al.* Ulcerative colitis is as common in Crete as in northern Europe: a 5-year prospective study. *Eur J Gastroenterol Hepatol* 1996; **8**: 893–898.

27 Nakamura Y, Labarthe DR. A case-control study of ulcerative colitis with relation to smoking habits and alcohol consumption in Japan. *Am J Epidemiol* 1994; **140**: 902–911.

28 Niewiadomski O, Studd C, Hair C, Wilson J, Ding NS, Heerasing N *et al.* Prospective population-based cohort of inflammatory bowel disease in the biologics era: Disease course and predictors of severity. *J Gastroenterol Hepatol* 2015; **30**: 1346–1353.

29 Ott C, Obermeier F, Thieler S, Kemptner D, Bauer A, Schölmerich J *et al.* The incidence of inflammatory bowel disease in a rural region of Southern Germany: a prospective population-based study. *Eur J Gastroenterol Hepatol* 2008; **20**: 917–923.

30 Parente JML, Coy CSR, Campelo V, Parente MPPD, Costa LA, Da Silva RM *et al.* Inflammatory bowel disease in an underdeveloped region of Northeastern Brazil. *World J Gastroenterol* 2015; **21**: 1197–1206.

31 Persson PG, Ahlbom A, Hellers G. Inflammatory bowel disease and tobacco smoke--a case-control study. *Gut* 1990; **31**: 1377–1381.

32 Radhakrishnan S, Zubaidi G, Daniel M, Sachdev GK MA. Ulcerative colitis in Oman: A prospective study of incidence and disease pattern from 1987 to 1994. *Diges* 1997; **58**: 266–270.

33 Ramadas A V., Gunesh S, Thomas GAO, Williams GT, Hawthorne AB. Natural history of Crohn’s disease in a population-based cohort from Cardiff (1986-2003): a study of changes in medical treatment and surgical resection rates. *Gut* 2010; **59**: 1200–1206.

34 Ranzi T, Bodini P, Zambelli A, Politi P, Lupinacci G, Campanini MC *et al.* Epidemiological aspects of inflammatory bowel disease in a north Italian population: a 4-year prospective study. *Eur J Gastroenterol Hepatol* 1996; **8**: 657–61.

35 Rodrigo L, Riestra S, Nino P, Cadahia V, Tojo R, Fuentes D *et al.* A population-based study on the incidence of inflammatory bowel disease in Oviedo (Northern Spain). *Rev Esp Enferm Dig* 2004; **96**: 296–305.

36 Romberg-Camps MJL, Hesselink-van de Kruijs MAM, Schouten LJ, Dagnelie PC, Limonard CB, Kester ADM *et al.* Inflammatory Bowel Disease in South Limburg (the Netherlands) 1991-2002: Incidence, diagnostic delay, and seasonal variations in onset of symptoms. *J Crohn’s Colitis* 2009; **3**: 115–124.

37 Sjöberg D, Holmström T, Larsson M, Nielsen AL, Holmquist L, Ekbom A *et al.* Incidence and natural history of ulcerative colitis in the Uppsala Region of Sweden 2005-2009 - Results from the IBD Cohort of the Uppsala Region (ICURE). *J Crohn’s Colitis* 2013; **7**: e351–e357.

38 Sjöberg D, Holmström T, Larsson M, Nielsen AL, Holmquist L, Ekbom A *et al.* Incidence and clinical course of Crohn’s disease during the first year - Results from the IBD Cohort of the Uppsala Region (ICURE) of Sweden 2005-2009. *J Crohn’s Colitis* 2014; **8**: 215–222.

39 Song EM, Lee H-S, Park SH, Kim GU, Seo MS, Hwang SW *et al.* Clinical Characteristics and Long-term Prognosis of Elderly-onset Ulcerative Colitis. *J Gastroenterol Hepatol* 2017. doi:10.1111/jgh.13826.

40 Tezel A, Dökmeci G, Eskiocak M, Ümit H, Soylu AR. Epidemiological features of ulcerative colitis in Trakya, Turkey. *J Int Med Res* 2003; **31**: 141–148.

41 Tozun N, Atug O, Imeryuz N, Hamzaoglu HO, Tiftikci A, Parlak E *et al.* Clinical characteristics of inflammatory bowel disease in Turkey: A multicenter epidemiologic survey. *J Clin Gastroenterol* 2009; **43**: 51–57.

42 Tragnone A, Hanau C, Bazzocchi G, Lanfranchi GA. Epidemiological characteristics of inflammatory bowel disease in Bologna, Italy--incidence and risk factors. *Digestion* 1993; **54**: 183–188.

43 Tsironi E, Feakins RM, Roberts CSJ, Rampton DS. Incidence of inflammatory bowel disease is rising and abdominal tuberculosis is falling in Bangladeshis in East London, United Kingdom. *Am J Gastroenterol* 2004; **99**: 1749–1755.

44 van der Heide F, Wassenaar M, van der Linde K, Spoelstra P, Kleibeuker JH, Dijkstra G. Effects of active and passive smoking on Crohnʼs disease and ulcerative colitis in a cohort from a regional hospital. *Eur J Gastroenterol Hepatol* 2011; **23**: 255–261.

45 Vegh Z, Burisch J, Pedersen N, Kaimakliotis I, Duricova D, Bortlik M *et al.* Incidence and initial disease course of inflammatory bowel diseases in 2011 in Europe and Australia: Results of the 2011 ECCO-EpiCom inception cohort. *J Crohn’s Colitis* 2014; **8**: 1506–1515.

46 Vind I, Riis L, Jess T, Knudsen E, Pedersen N, Elkjær M *et al.* Increasing incidences of inflammatory bowel disease and decreasing surgery rates in Copenhagen City and County, 2003-2005: A population-based study from the Danish Crohn colitis database. *Am J Gastroenterol* 2006; **101**: 1274–1282.

47 Vuceljć B, Korać B, Sentić M, Millličlć D, Hadžić N, Jureša V *et al.* Ulcerative colitis in Zagreb, Yugoslavia: Incidence and prevalence 1980-1989. *Int J Epidemiol* 1991; **20**: 1043–1047.

48 Yamamoto-Furusho JK. Clinical Epidemiology of Ulcerative Colitis in Mexico. *J Clin Gastroenterol* 2009; **43**: 221–224.

49 Yang H, Li Y, Wu W, Sun Q, Zhang Y, Zhao W *et al.* The incidence of inflammatory bowel disease in Northern China: A prospective population-based study. *PLoS One* 2014; **9**: 5–10.

50 Yapp TR, Stenson R, Thomas GA, Lawrie BW, Williams GT HB. Crohn’s disease incidence in Cardiff from 1930: an update for 1991-1995.pdf. *Eur J Gastroenterol Hepatol* 2000; **12**.https://journals.lww.com/eurojgh/Abstract/2000/12080/Crohn_s_disease_incidence_in_Cardiff_from_1930__an.10.aspx.

51 Zahedi MJ, Darvish Moghadam S, Hayat Bakhsh Abbasi M, Dehghani M, Shafiei Pour S, Zydabady Nejad H *et al.* The incidence rate of inflammatory bowel disease in an urban area of iran: a developing country. *Middle East J Dig Dis* 2014; **6**: 32–6.

52 Zeng Z, Zhu Z, Yang Y, Ruan W, Peng X, Su Y *et al.* Incidence and clinical characteristics of inflammatory bowel disease in a developed region of Guangdong Province, China: A prospective population-based study. *J Gastroenterol Hepatol* 2013; **28**: 1148–1153.

53 Zhai H, Huang W, Liu A, Li Q, Hao Q, Ma L *et al.* Current smoking improves ulcerative colitis patients’ disease behaviour in the northwest of China. *Gastroenterol Rev* 2017; **4**: 286–290.

54 Zhao J, Ng SC, Lei Y, Yi F, Li J, Yu L *et al.* First prospective, population-based inflammatory bowel disease incidence study in mainland of China: The emergence of ‘western’ disease. *Inflamm Bowel Dis* 2013; **19**: 1839–1845.
